# A Rare Case of Primary CNS Lymphoma in an HIV-Positive Patient Mimicking CNS Tuberculosis

**DOI:** 10.7759/cureus.62426

**Published:** 2024-06-15

**Authors:** Nurafiqah Farhana Muhd Yazid, Mohamad Izzat Arslan Che Ros, Shahrunizam Awang Setia

**Affiliations:** 1 Internal Medicine, Universiti Sains Islam Malaysia, Nilai, MYS; 2 Department of Radiology, Universiti Kebangsaan Malaysia Medical Centre, Kuala Lumpur, MYS; 3 Department of Radiology, University Malaysia Sabah, Kota Kinabalu, MYS

**Keywords:** tuberculosis, tuberculoma, hiv, cerebral toxoplasmosis, diffuse large b-cell lymphoma

## Abstract

Primary cerebral lymphoma in immunocompromised patients is rare and challenging to diagnose. Its presentation can have similarities with other opportunistic diseases like cerebral toxoplasmosis and tuberculoma, to name a few, which may affect the subsequent management. Here, we report a case of a gentleman with human immunodeficiency virus (HIV) who presented with clinical features of fever, confusion, and generalized lethargy. His imaging features mimicked those of central nervous system (CNS) tuberculosis (TB), and he was treated for one. Unfortunately, the patient failed to respond to the anti-tuberculosis treatment and continued to deteriorate eventually succumbing to his illness. Brain histopathology biopsy confirmed the diagnosis of diffuse large B-cell lymphoma. We aim to illustrate the importance of a high index of suspicion with timely action taken whenever the anticipated finding or response to treatment is not observed. Multiple imaging modalities coupled with biochemistry and histopathological investigations should be considered in discriminating competing diagnoses.

## Introduction

Primary central nervous system lymphoma (PCNSL) is a rare extra-nodal non-Hodgkin lymphoma (NHL) accounting for 1-3% of all NHL and 3-5% of all primary brain tumors [1]. Diagnostic biopsy of brain lesions is the gold standard as it is safe and effective [2]. It is generally recommended following the failure of empirical treatment. However, for unstable patients requiring urgent treatment, we still rely heavily on brain imaging to aid diagnosis. Prompt diagnosis is challenging since its clinical and imaging features can mimic other infections, namely, CNS tuberculosis (TB) [3,4]. We report a histopathologically confirmed case of human immunodeficiency virus (HIV)-related PCNSL mistaken for CNS TB. We aim to highlight the similarities of clinical and imaging features of PCNSL with CNS TB, hence the importance of high clinical suspicion.

## Case presentation

Our patient is a 38-year-old gentleman with underlying bronchial asthma. He first presented to us with a two-week history of fever, generalized lethargy, and confusion. He also had a prolonged cough for one month associated with loss of appetite and weight loss. Clinically, he was confused, but obeying commands, lethargic looking. There was also the presence of oral candidiasis. Auscultation of the lungs revealed reduced air entry over the left side. Neurological examination showed hyperreflexia over the left limbs with a Medical Council Research (MRC) power scale of 4/5 over the left upper limb. Basic investigation showed normal hemoglobin of 14.5g/dL (13-17), white cell count of 4.5 x 109/L, and platelet of 259 x 103 µL (150-450 x 103 µL), and both of his renal and liver function tests were also normal. He was initially treated for pneumonia, and his HIV RNA PCR was found to be positive. 

**Figure 1:**
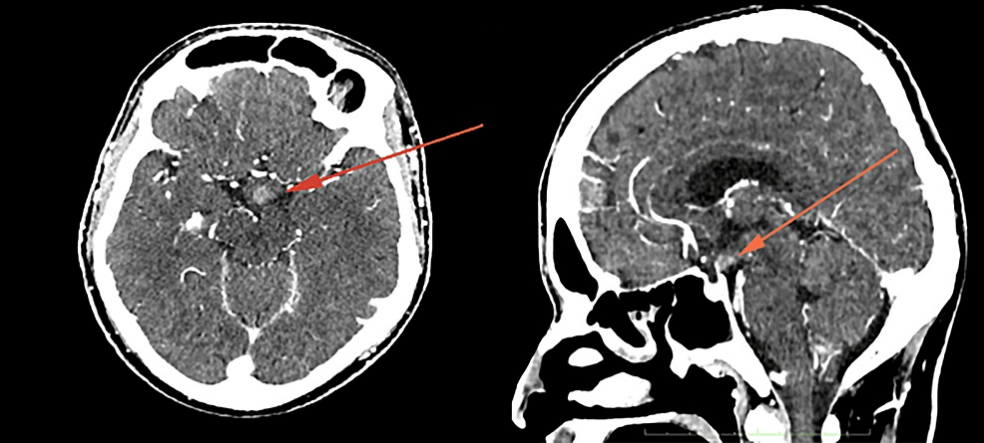
Enhancing hypothalamic lesion noted in the same contrasted CT brain (arrow) along with the left periventricular rim enhancing lesion.

**Figure 2:**
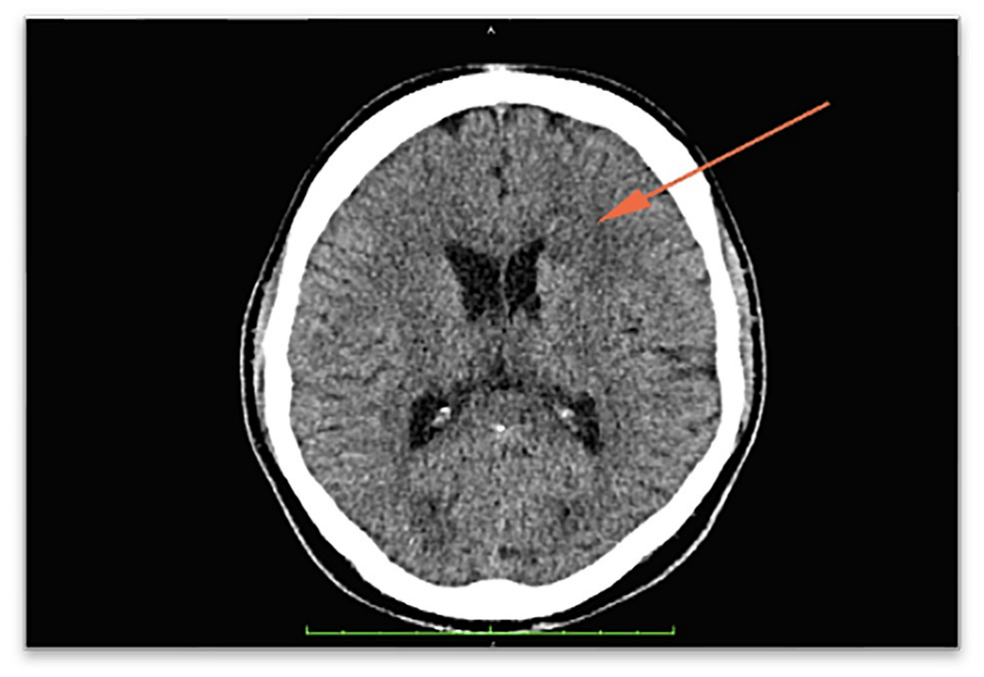
Non-enhanced CT brain showed an ill-defined hypodensity over the left frontal white matter (arrow).

**Figure 3:**
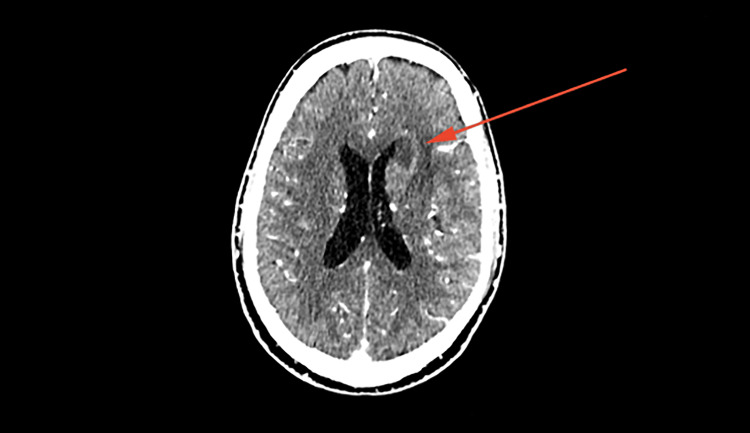
Contrasted CT brain showed solitary rim enhancing lesion at left frontal periventricular (arrow) with the mass effect onto the left frontal horn of the ventricle.

The initial computed tomography (CT) brain scan revealed an ill-defined white matter hypodensity in the frontal region (Figure 1), exhibiting rim enhancement post-administration of gadolinium (Figure 2). There was also an enlarged hypothalamus with an enhancing hypothalamic lesion (Figure 3). Considering the underlying HIV, these findings raise a few differential diagnoses, such as infective process or granulomatous infection, toxoplasmosis, primary CNS lymphoma, or CNS TB, while the hypothalamic lesion may be a separate neoplastic lesion. His cerebral spinal fluid (CSF) analysis showed a high protein of 2,321 mg/L (150-600 mg/L) and low glucose of 1.13 mmol/L ( 2.77-4.44 mmol/L) with no pleocytosis. CSF Indian ink was negative, and there was no growth in both CSF and peripheral blood cultures. CSF fluids were also tested for various antigens, including Haemophilus influenzae B, Group B *Streptococcus*, *Streptococcal pneumoniae*,* Escherichia coli, * and *Neisseria meningitides,* which all came back negative. Other serological investigations sent were *Toxoplasmosis* IgG, which was non-reactive. Cytomegalovirus (CMV) IgG returned reactive with cytomegalovirus IgM non-reactive indicating a possible past CMV infection. His CD4 count was only 16 cells/uL.

**Figure 4:**
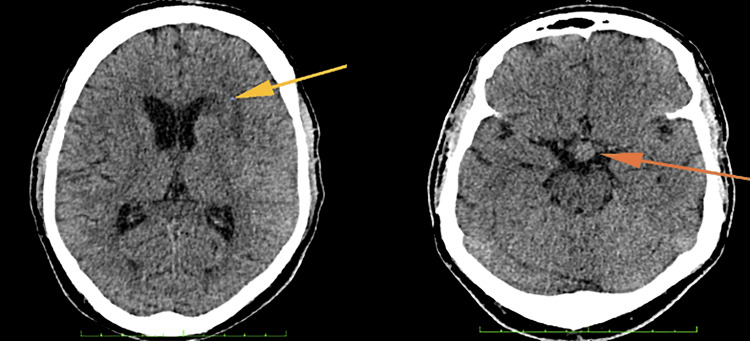
Similar left frontal lesion (yellow arrow) with a larger hypothalamic lesion (orange arrow). No intracranial bleeding.

He was then started on a combination of anti-tuberculosis treatments consisting of EHRZ (ethambutol, isoniazid, rifampicin, and pyrazinamide) after a high suspicion of CNS TB along with intravenous (IV) dexamethasone 4 mg tds. After a few days in the ward, his Glasgow Coma Scale (GCS) dropped from 13 to 10. A repeated non-contrasted CT brain showed the development of early non-obstructive hydrocephalus with a comparatively larger left frontal lesion (Figure 4). 

**Figure 5:**
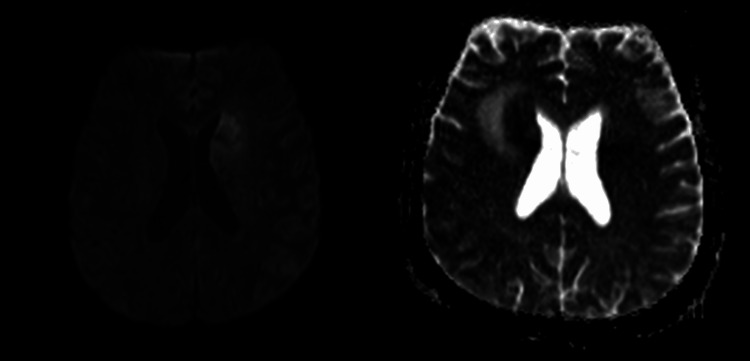
Left frontal lesion demonstrating restricted diffusion on Diffusion‐weighted imaging (DWI)/apparent diffusion coefficient (ADC).

**Figure 6:**
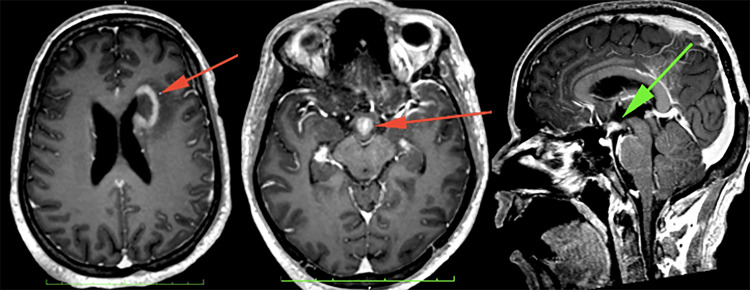
Multiple nodular enhancing lesions in the hypothalamus (orange arrow, middle image, and green arrow). Noted that there was also a nodular enhancement along both hippocampi (middle image).

**Figure 7:**
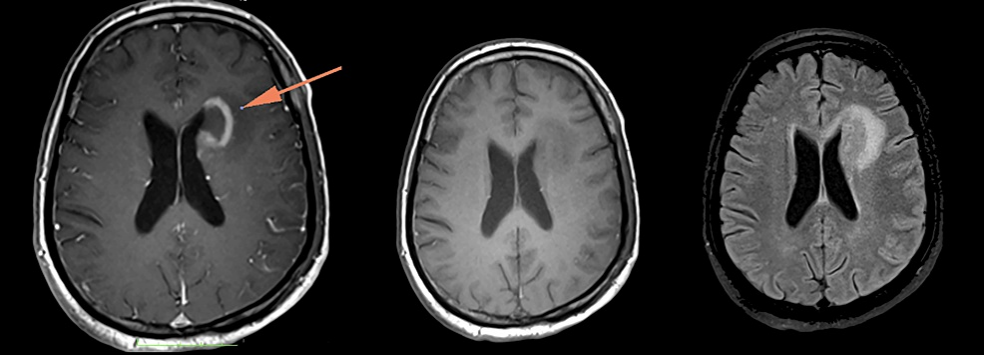
Rim-enhancing lesion in the left frontal periventricular region (orange arrow), which demonstrates a low signal in T1W and a high signal in T2W.

**Figure 8:**
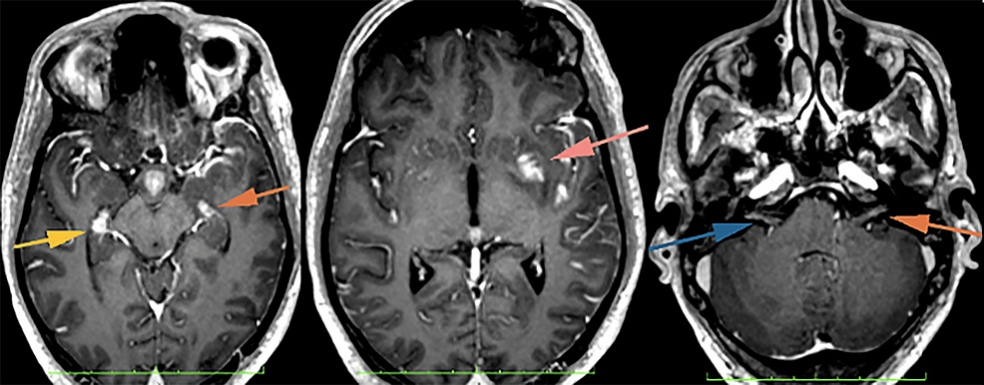
Nodular enhancement along both hippocampi (left image), left lentiform nucleus (middle image), and bilateral intra-canalicular of the seventh and eighth cranial nerves (right image).

**Figure 9:**
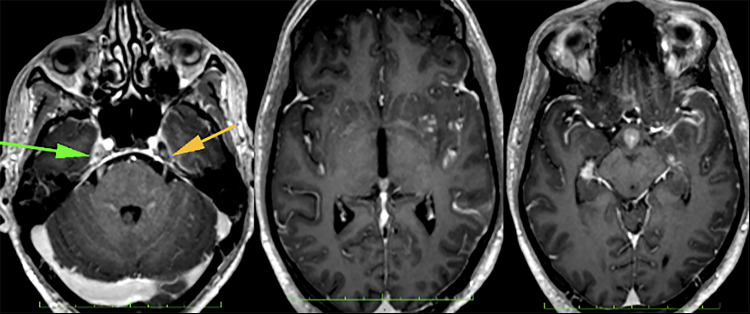
There was also nodular enhancement involving the bilateral trigeminal nerve. The right trigeminal nerve is also enlarged predominantly at the right Meckel Cave (green arrow).

**Figure 10:**
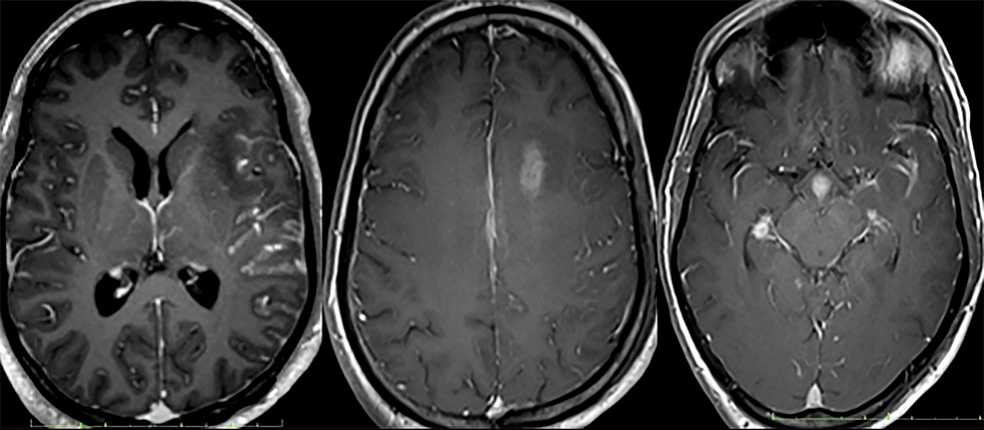
Leptomeningeal enhancement over the left frontotemporoparietal, bilateral parasagittal, and basal cisterns.

**Figure 11:**
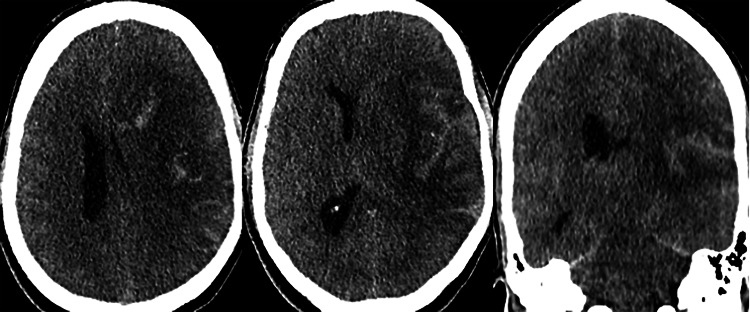
New subarachnoid and intraparenchymal hemorrhage with a worsening mass effect, cerebral edema, and hydrocephalus.

He then had a magnetic resonance imaging (MRI) brain done that showed a formation of the left periventricular abscess and diffuse nodular leptomeningeal enhancement (Figures 5-10). Non-pyogenic infections such as tuberculosis or fungal-like cryptococcus were among the prime differential diagnoses. Unfortunately, he had another drop in GCS needing intubation to secure his airway. Repeated non-contrasted CT brain showed a new subarachnoid and intraparenchymal hemorrhage with a worsening mass effect, cerebral edema, and hydrocephalus (Figure 11).

As his condition continued to deteriorate, he underwent craniectomy and lobectomy, and samples were collected for brain histopathological examination (HPE) and culture. He succumbed to his illness with the cause of death of TB meningitis and intracranial hemorrhage. The histopathology report later came back as diffuse large B-cell lymphoma, positive for CD20 and CD79a.

## Discussion

Progressive immunosuppression in HIV-positive patients may cause an increase in their susceptibility to several opportunistic infections affecting the CNS. They are predisposed to AIDS-defining diseases. In this population, the most common etiology of brain lesions includes cerebral toxoplasmosis, progressive multifocal leukoencephalopathy (PML), and primary central nervous system lymphoma (PCNSL), followed by tuberculoma infrequently [5].

PCNSL is relatively rare accounting for approximately 3-5% of all primary brain tumors. The majority of intracerebral lymphomas are non-Hodgkin's lymphomas, and approximately 95% are diffuse large B-cell lymphomas [1]. Due to the increasing prevalence of HIV infection and the growing number of organ transplantations, the incidence of PCNSL has been increasingly observed in the immunocompromised population. Typically, patients can come with confusion, lethargy, memory loss, hemiparesis, or seizures. These symptoms can progress rapidly, and the focal neurological deficit largely depends on the location of the lesion. Contrarily, the occurrence of systemic "B" symptoms like weight loss, fever, and night sweats are rare. Our patient did have some of these features, and his CD4 counts of 16 cells/microliters were also typical of those predisposed to this condition. However, clinical symptoms alone may not be sufficient to reach a concrete diagnosis, hence the need for other modalities. There is a stark distinction in the typical imaging characteristics of PCNSL between immunocompetent patients and those who are immunocompromised. In immunocompromised patients, PCNSL is commonly multifocal; tends to be larger, with rapid progression in size; and demonstrates irregular and nodular ring enhancement with a necrotic center, and intra-tumoral hemorrhages may be present. These features are less likely to occur among immunocompetent patients [6]. Epstein-Barr virus (EBV) deoxyribonucleic acid (DNA) detection from cerebrospinal fluid (CSF) samples is quite sensitive for PCNSL but was not sent in this case.

It was estimated that 164,000 adults contracted CNS TB worldwide in 2019, of which 23% were HIV-positive. Seventy percent of global CNS TB incidence occurred in Southeast Asia and Africa [7]. Given the familiarity of CNS TB in our region, the diagnosis was at the forefront of our considerations. Empirical treatment with anti-TB medications was initiated after considering his clinical symptoms of prolonged cough with consolidation on his chest radiograph, along with all of his brain imaging features. This decision is consistent with the currently available guidelines and proposed algorithm on the approach to intracranial mass lesions in an HIV-positive patient [8,9]. However retrospectively, none of his sputum and CSF samples were positive for *Mycobacterium*. In CNS tuberculosis, the most common imaging finding is a leptomeningeal enhancement, in which most patients would also have concurrent pulmonary/military TB. Subpial exudate is primarily seen in the inferomedial surface of the frontal, anteromedial surface of temporal lobes, the superior aspect of the cerebellum, the floor of the third ventricle that then goes into the interpeduncular cistern, and the anterior part of pontomesencephalic cisterns. The enhancement is usually thick exudates with nodular patterns. Other common presentation includes tuberculoma, tuberculous abscess, and encephalopathy from cerebritis [10]. These overlapping imaging features in our patient had made it challenging to come up with a prompt and accurate diagnosis. Other than the ring-enhancing lesion that is very non-specific, an additional aspect to consider when narrowing down the diagnosis is the pattern of leptomeningeal enhancement. Leptomeningeal carcinomatosis, lymphoma, and metastases often demonstrate a focal type of enhancement, whereas the enhancement pattern is commonly diffuse in TB meningitis. *Cryptococcus* infection and pyogenic infection will usually show diffuse types of leptomeningeal enhancement. In our particular case, the imaging features indicate a typical granulomatous process with prominent thick leptomeningeal enhancement in the basal cisterns. However, this is not always the scenario. The pattern of cranial nerve enhancement seen on MRI with the respective possible differential diagnoses is summarized in Table 1.

**Table 1:** Differential diagnosis of cranial nerve hypertrophy and enhancement. Citation: Saremi et al. [11]

Disease	Associated pattern
Metastases, lymphoma, leptomeningeal carcinomatosis	Enhancing and usually associated with hypertrophy, due to perineural spread
TB leptomeningeal	Occurs due to vascular compromise, ischemia, or nerve entrapment by the exudates or tuberculomas especially at its cisternal segment. Most commonly affects CN II, III, IV, VI, and VII.
Others: neurosarcoidosis, amyloid neuropathy, neurosyphilis, demyelinating polyneuropathy, neurofibromatosis-1	Nodular, nerve enlargement

The rapid decline in our patient's condition in less than two weeks may suggest a higher likelihood of an infectious process rather than tumor progression. However, it is worth noting that PCNSL is an aggressive malignancy with a reported overall survival of approximately 1.5 to three months if left untreated [12]. The hallmark in this case leading to the patient's demise was the development of a spontaneous intracerebral hemorrhage. A population-based cohort study among HIV-positive patients has demonstrated higher rates of spontaneous intracranial hemorrhage with a hazard ratio of 3.28 (95% confidence interval (CI) 1.75-6.12), with the effect even increasing to 7.64 (95% CI 3.78-15.43) in those with AIDS-defining conditions [13]. Apart from AIDS-defining conditions, some risk factors that may lead to spontaneous ICB in HIV-positive patients include substance abuse and concurrent hepatitis C infection. On the other hand, we do observe on some rare occasions an intracerebral or intraventricular hemorrhage related to CNS tuberculosis potentially attributed to bleeding of inflammatory vessels. While it is uncommon, intra-tumoral bleeding resulting from high immunoreactivity for vascular endothelial growth factor in PCNLS has been documented in certain literature [14]. 

Another modality that may aid in diagnosis is MR spectroscopy, where increased lipid resonance is the most specific finding for PCNSL. Harting et al. discovered a significantly higher number of lipid peaks in solid PCNSL compared to solid low and high-grade astrocytomas. This is due to cell death resulting in abundant macrophages and the multiplication of membrane components turnover in transformed lymphoid cells [6]. In tuberculoma, the lipids are typically more elevated than the lactate. There may also be a role of positron-emitted tomography (PET) CT in detecting PCNSL as it shows intense uptake of 18-fluorodeoxyglucose (FDG); however, it is still not clearly defined. It can also help to differentiate PCNSL from toxoplasmosis in immunocompromised patients as decreased FDG is seen in toxoplasmosis compared to high uptake in PCNSL. However, in cases with atypical radiological findings on MRI in the AIDS setting, FDG-PET has not particularly been shown to aid localization or diagnosis [15].

## Conclusions

TB and lymphoma are notorious as great mimickers in the world of medicine, posing a considerable challenge to diagnose and manage especially as they can also co-exist. We have discussed an atypical presentation of PCNSL in an HIV-positive patient with overlapping clinical and imaging features who was initially diagnosed with CNS TB. Multiple imaging modalities coupled with biochemistry and histopathological investigations should be employed in discriminating competing diagnoses. Whenever the expected outcome is not reached, it is important to consider alternative diagnoses particularly in immunocompromised individuals to facilitate an accurate and timely treatment to improve patient outcomes in such challenging cases.
